# Editorial: Secondary Metabolism. An Unlimited Foundation for Synthetic Biology

**DOI:** 10.3389/fmicb.2015.01562

**Published:** 2016-01-11

**Authors:** Ana Lúcia Leitão, Francisco J. Enguita

**Affiliations:** ^1^MEtRICs, Departamento de Ciências e Tecnologia da Biomassa, Faculdade de Ciências e Tecnologia, Universidade NOVA de LisboaCaparica, Portugal; ^2^Faculdade de Medicina, Instituto de Medicina Molecular, Universidade de LisboaLisboa, Portugal

**Keywords:** synthetic biology, filamentous bacteria, fungi, algae, secondary metabolism, metabolic engineering

The foundations of synthetic biology rely on the concept that all living systems are constituted by functional and structural modules that could be rationally engineered to build organisms that are not easily generated by evolution. However, the cellular complexity and metabolic diversity found in organisms present both a significant challenge and opportunity to foster this interdisciplinary scientific area. Secondary metabolism is a group of biological processes which are dispensable for cell growth. In microorganisms such as fungi and filamentous bacteria, secondary metabolism is an important source of biologically active compounds (Leitão and Enguita, 2014). The genes encoding for the enzymes involved in secondary metabolism are frequently clustered together displaying a modular organization, constituting a perfect target for the application of synthetic biology principles with an almost unlimited potential for the construction of new cell factories, which is mainly constrained by the knowledge of the working rules of the functional blocks governing the biosynthesis of a particular metabolite.

More than 35 years after the first seminal ideas by Hopwood, who hypothesized about the possibility of a rational design of new antibiotics based on the combination of biosynthetic genes (Hopwood and Chater, 1980), secondary metabolites are gaining a new relevance. Modern genetic techniques have enabled complete genome sequences of secondary metabolite producers to be thoroughly and rapidly interrogated to uncover loci relevant for metabolite biosynthesis. The developed genome editing and engineering techniques are paving the way for rational pathway design and construction, modifying a genotype with the main objective of giving rise to a desired phenotype (Paddon et al., 2013; Paddon and Keasling, 2014; Hoynes-O'connor and Moon, 2015). Recent applications of genome editing techniques such as TALE nucleases or CRISPR-Cas9 in the rational engineering of primary and secondary metabolites are an excellent example of the potential in this area (Huang et al., 2015; Lv et al., 2015; Wu et al., 2015).

This Frontiers research topic was proposed to compile new trends for the application of synthetic biology methods and protocols to the field of secondary metabolites. We are extremely grateful to all the authors that have contributed to enrich the contents of the research topic. The topic contributions include six articles, covering wide aspects of secondary metabolism in organisms ranging from filamentous bacteria to microalgae.

Two excellent articles by Mattern et al. and Chavez et al. describe applications of synthetic biology principles for the manipulation of secondary metabolite biosynthesis in filamentous fungi. Interestingly, the article by Chavez et al. also explore the idea of genome mining to search and explore new metabolites in filamentous fungi isolated from extreme environments.

Several practical applications of synthetic biology principles were discussed by Giessen and Marahiel. In this article, the authors present a large repository of modular catalytic enzymes able to modify cyclic dipeptides, and demonstrate how combinatorial chemistry can be applied to custom design new molecules with improved biological activities. Moreover, the work by Gressler et al. also included in the research topic, describes a high-performance system for heterologous expression in *Aspergillus terreus* based on the *tet*A promoter and the DNA-binding domain of its cognate transcription factor TetR. As a proof of concept, the authors used the system to express the polyketide synthase-encoding gene *osr*A, and demonstrated its catalytic activity. Other interesting topic discussed by Beites and Mendes reflects the increasing number of genomic data generated by the bacterial genome sequencing projects which can be further exploited as an unlimited source of metabolic diversity. In fact, following the author's ideas, several biosynthetic clusters for secondary metabolites present reduced or no expression (also known as “cryptic” clusters), constituting a pool of novel metabolites waiting to be mined and characterized. Within this context, the article described rational strategies and tools to be used in the development of chassis structures for the heterologous expression of biosynthetic gene clusters in filamentous bacteria. Finally, the topic also includes an article by Gimpel et al. which discusses the strategies for rational metabolic engineering of microalgae, a very promising tool for the production of several products including biofuels, carotenoids, and bio-hydrogen.

We hope that this research topic will be interesting and useful to the general audience of Frontiers in Microbiology journal, and that the treated topics act as crystallization nuclei for new ideas and future applications of the principles of synthetic biology to the design of new secondary metabolites with improved biological activities.

## Conflict of interest statement

The authors declare that the research was conducted in the absence of any commercial or financial relationships that could be construed as a potential conflict of interest.
